# The Promise of Combination Therapies with FOXM1 Inhibitors for Cancer Treatment

**DOI:** 10.3390/cancers16040756

**Published:** 2024-02-12

**Authors:** Nawal Merjaneh, Mona Hajjar, Ying-Wei Lan, Vladimir V. Kalinichenko, Tanya V. Kalin

**Affiliations:** 1 Center for Cancer and Blood Disorders, Phoenix Children’s Hospital, Phoenix, AZ 85016, USA; 2 Department of Child Health, Division of Hematology and Oncology, The University of Arizona College of Medicine-Phoenix, Phoenix, AZ 85004, USA; 3 The Columbian College of Arts and Sciences, George Washington University, Washington, DC 20052, USA; hajjarmona1@gmail.com; 4 Phoenix Children’s Research Institute, The University of Arizona College of Medicine-Phoenix, Phoenix, AZ 85004, USA; yingweilan@arizona.edu (Y.-W.L.);; 5 Division of Neonatology, Phoenix Children’s Hospital, Phoenix, AZ 85016, USA

**Keywords:** combination therapies, FOXM1 inhibitor, cancer

## Abstract

**Simple Summary:**

FOXM1 is an oncogenic transcription factor that has been implicated in cancer progression, metastases, and chemotherapy resistance. Multiple small-molecule FOXM1 inhibitors have been studied in the lab, but none have made it to clinical trials. The aim of this review is to describe the studied combination therapies with FOXM1 inhibitors. We evaluated the synergistic role of FOXM1 inhibitors with chemotherapy and molecular-targeted therapies for cancer treatment. Small-molecule FOXM1 inhibitors are promising compounds whose therapeutic benefits can be applied to different pediatric and adult cancers.

**Abstract:**

Forkhead box M1 (FOXM1) is a transcription factor in the forkhead (FOX) family, which is required for cellular proliferation in normal and neoplastic cells. FOXM1 is highly expressed in many different cancers, and its expression is associated with a higher tumor stage and worse patient-related outcomes. Abnormally high expression of FOXM1 in cancers compared to normal tissue makes FOXM1 an attractive target for pharmacological inhibition. FOXM1-inhibiting agents and specific FOXM1-targeted small-molecule inhibitors have been developed in the lab and some of them have shown promising efficacy and safety profiles in mouse models. While the future goal is to translate FOXM1 inhibitors to clinical trials, potential synergistic drug combinations can maximize anti-tumor efficacy while minimizing off-target side effects. Hence, we discuss the rationale and efficacy of all previously studied drug combinations with FOXM1 inhibitors for cancer therapies.

## 1. Introduction

Forkhead box M1 (FOXM1) protein is a member of the forkhead box (FOX) transcription factor family that shares homology in the Winged Helix/*Forkhead* DNA-binding domain [1,2,3]. FOXM1 is highly expressed during normal embryogenesis and is extinguished in terminally differentiated cells [4]. Homozygous deletion of *Foxm1* in mice is lethal; embryos die in utero between 13.5 and 17.5 days of gestation due to severe proliferation defects in multiple organs, including the heart, liver, and blood vessels [5,6]. Conditional deletion of *Foxm1* in various mice cell types inhibits cell proliferation [4,7,8,9,10,11,12,13], whereas the overexpression of *Foxm1* accelerates cell proliferation [14,15,16,17] and prevents age-related defects in cell cycle progression [18]. FOXM1 also regulates the inflammatory response and intracellular metabolic processes [19,20,21,22,23,24,25]. Consistent with the important role of FOXM1 in cell cycle progression, FOXM1 expression is increased during carcinogenesis [26]. The human *FOXM1* gene is located on the chromosomal band 12p13 [27], which is frequently amplified in different cancers, including prostate cancer [28,29], breast adenocarcinoma [30], head and neck squamous cell carcinoma [31], nasopharyngeal carcinoma [32], and cervical squamous carcinoma [33]. The increased level of FOXM1 in different types of cancer induces cancer progression, invasion, metastasis, and tumor-associated angiogenesis [34,35,36]. The differential expression of FOXM1 in tumors compared to normal tissues makes it an attractive target for pharmacological inhibition [26,37]. Several FOXM1 inhibitors have been evaluated in pre-clinical studies. However, no compound has been advanced to clinical trials. Several natural products like honokiol, curcumin, genistein, solanum incanum extract, and diarylheptanoids were found to decrease the expression of FOXM1 and its target genes [38,39,40,41] or attenuate the *FOXM1* gene network [42]. Cellular-based in vitro assays uncovered the FOXM1-inhibitory activities of thiazole antibiotics, including Siomycin A and thiostrepton [43]. It was shown that the anti-FOXM1 activity of these compounds was executed through proteasome inhibition. Furthermore, pharmacological proteasome inhibitors, such as bortezomib and carfilzomib, inhibited FOXM1 activity to the same level [44]. However, these FOXM1 inhibitors are not specific to FOXM1 and may carry severe off-targeted side effects [45]. Therefore, there has been a movement to identify compounds that can specifically bind and inhibit FOXM1. Among them are RCM-1, STL427944, and STL001, representing the small molecules that inhibit FOXM1 nuclear translocation and induce its cytoplasmic degradation [46,47,48,49] (Table 1). RCM-1 has shown excellent anti-tumor activity against different tumor cell lines and in mouse tumor xenografts without observed toxic side effects [47,50]. One mechanism by which RCM-1 inhibits FOXM1 is the disruption of protein–protein interactions between FOXM1 and β-catenin, a key receptor of the canonical Wnt signaling pathway. The inhibition of FOXM1–β-catenin interactions by RCM-1 results in the degradation of both proteins, leading to a robust anti-tumor effect [47]. Other specific FOXM1 inhibitors, such as FDI-6 and XST-20, bind to the FOXM1 DNA-binding domain, which subsequently prevents FOXM1 interaction with DNA and decreases FOXM1 transcriptional activity without a decrease in protein level [51,52]. However, targeting a single oncogenic pathway may result in acquired resistance and prevent a durable anti-tumor response. Thus, we discuss here the rationale for using FOXM1 inhibitors in combination with cytotoxic or other targeted therapies for the treatment of different cancers (Table 1).

### 1.1. FOXM1 Inhibitors in Combination with Cytotoxic Chemotherapy

**Figure 1:**
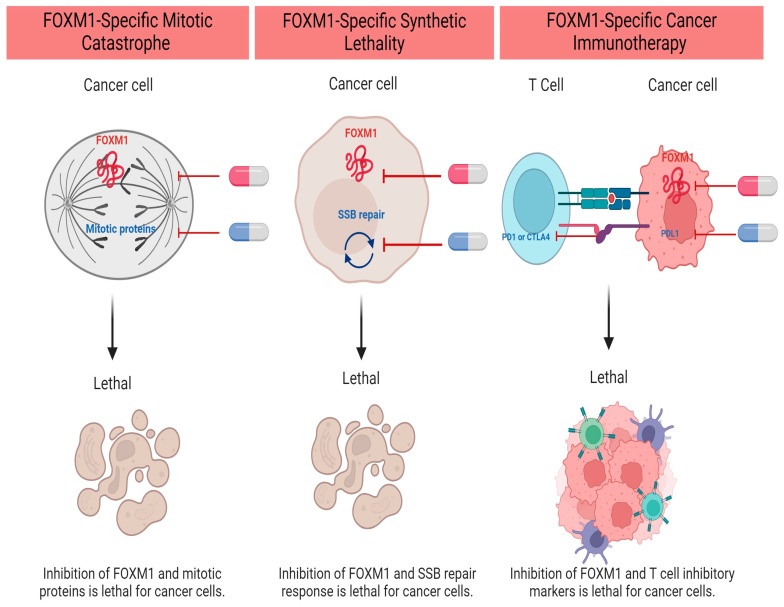
FOXM1 Inhibitor-Based Combination Therapies in Upfront Regimens. Created with BioRender.com (accessed on 5 December 2023).

Cytotoxic chemotherapy is still the cornerstone for the management of most pediatric and adult malignancies. They are divided into categories based on their mechanism of action. Multiagent chemotherapy is frequently used to overcome cancer intrinsic resistance (Goldie–Coldman hypothesis). However, refractory and relapsed tumors are often encountered in clinical practice, with very few options left for salvage treatment. While chemotherapy resistance is often multifactorial, FOXM1 overexpression has been repeatedly observed in many resistant solid tumors [73,74]. Because FOXM1 has a critical role in DNA repair after cell exposure to DNA-damaging agents, its expression in resistant cells is a defensive mechanism to escape cell death. FOXM1 regulates the transcription of multiple DNA damage repair (DDR) proteins and enhances DNA single- and double-strand break repair [73]. Furthermore, FOXM1 protein levels correlate with genomic instability and aneuploidy [26,75], and chromosomal instability is frequently linked to chemotherapy resistance and poor patient prognosis [76,77]. Collectively, FOXM1 is an attractive therapeutic target to be considered as an addition to chemotherapy to improve outcomes and prevent chemotherapy-resistant tumors (Figure 1).

#### 1.1.1. Combination with Alkylating Agents

a. Platinum analogs: Cisplatin and carboplatin work by forming DNA adducts that disrupt DNA structure, leading to irreparable damage and apoptosis initiation. Cisplatin-resistant ovarian and oral carcinoma cells express a higher level of FOXM1 [78,79]. Not surprisingly, FOXM1 overexpression correlates with the expression of multiple DNA damage response proteins such as BRCA2, XRCC1, and EXO1 [74,80], likely enhancing the efficiency of DNA repair and maintaining cell survival. FOXM1 also induces β-catenin expression, nuclear localization, and activation, promoting the epithelial-to-mesenchymal transition (EMT) and stem cell phenotype in ovarian cancer cells [78]. Concurrent treatment with cisplatin and a FOXM1 inhibitor restores cisplatin’s anti-tumor activity. Combination treatment exhibits an enhanced proapoptotic activity in contrast to a single agent in ovarian cancer and oral squamous cell carcinoma xenografts [78,79].

b. Temozolomide (TMZ) is an alkylator that breaks DNA into double-strand DNA fragments. It is widely used for the treatment of high-grade gliomas. However, resistance to TMZ is usually inescapable and correlates with worse survival outcomes. Multiple studies demonstrated an increased FOXM1 level in TMZ-resistant cells [61,81,82]. FOXM1 expression promotes DNA repair via the upregulation of RFC5 and Rad 51 proteins [81,82]. FOXM1 also upregulates the expression of the antiapoptotic protein Survivin, which has been linked to TMZ resistance [61]. Collectively, concurrent treatment with thiostrepton or bortezomib and TMZ restores TMZ sensitivity and intensifies its apoptotic activity.

#### 1.1.2. Combination with Topoisomerase II Inhibitors

Anthracyclines inhibit the topoisomerase II enzyme, intercalate between DNA bases, and cleave DNA into fragments. The accumulation of double-strand DNA breaks overwhelms the DNA repair response and drives the cells into apoptosis [83]. In anthracycline-resistant breast cancer cells, FOXM1 is highly upregulated [84]. FOXM1 overexpression is associated with an increase in DNA damage response proteins such as ATM and NBS1 [85,86]. It is also associated with the upregulation of antiapoptotic genes such as XIAP and Survivin [87]. Ghandhariyoun et al. demonstrated that FOXM1 aptamer enhanced doxorubicin-induced apoptosis in breast cancer cells and mouse xenografts [53]. Furthermore, thiostrepton increased doxorubicin accumulation in Jurkat cells due to the suppression of glutathione S-transferase pi (GSTpi) expression, a known culprit in multidrug resistance [88].

#### 1.1.3. Combination with Mitotic Spindle Inhibitors

a. Vinca alkaloids: Vincristine, vinblastine, and vinorelbine are tubulin inhibitors. They inhibit microtubule formation and lead to cell cycle arrest at mitosis. Donovan et al. demonstrated that the combination therapy of RCM-1 and VCR exhibited superior anti-tumor activity in contrast to single-agent therapy. The authors explored using a lower VCR dose to limit VCR-induced neuropathy and liver dysfunction while maintaining anti-tumor activity in rhabdomyosarcoma cell lines and mouse xenografts [50]. Interestingly, RCM-1 can be injected intravenously using tumor-specific nanoparticles. Nanoparticle-based drug delivery enables more targeted and effective drug delivery and opens the door for anti-cancer combination therapies in a single infusion [50]. We previously showed that RCM1 treatment increases the duration of mitosis in tumor cells [47], rationalizing the use of RCM1 with mitotic inhibitors to increase the efficacy of anti-cancer therapy.

b. Taxanes: Paclitaxel and docetaxel are similar to vinca alkaloids in that they inhibit tubulin and induce mitosis arrest. FOXM1 has an essential role in mitotic spindle formation, chromosome alignment, segregation, and daughter cell formation. Depletion of FOXM1 by thiostrepton (TST) downregulates the expression of the kinesin protein KIF20A, mediating mitotic spindle dysfunction and cellular senescence [74]. In pancreatic cancer cells, TST inhibits the prohibin1 protein, decreasing the phosphorylated ERK1/2 level, and decreases the expression of the ABC drug transporter, fostering a higher intracellular anti-cancer drug concentration [62]. Hence, TST synergizes with microtubule inhibitors, such as paclitaxel, to overcome drug resistance and induce mitotic catastrophe [62,63].

#### 1.1.4. Combination with Antimetabolites

The antimetabolite family represents a large group of anti-cancer therapies, including folic acid antagonists and purine and pyrimidine analogs. They disturb DNA synthesis through the inhibition of key molecules in DNA’s structure.

a. Fluorouracil (5-FU) is a pyrimidine analog that blocks DNA synthesis through the suppression of the thymidylate synthase enzyme (TYMS) and the depletion of thymidine triphosphate. 5-FU is commonly used in adult solid tumors, including pancreatic and colon cancers. 5-FU-resistant colon cancer and cholangiocarcinoma cells exhibit high levels of FOXM1 and TYMS [64,65]. Moreover, FOXM1 binds directly into the TYMS promotor region and induces its expression [64]. Hence, FOXM1 overexpression mediates 5-FU resistance because of the increase in drug targets [64]. In addition, FOXM1 overexpression induces ABCC10 expression and increases drug efflux, promoting 5-FU resistance because of the decrease in the intracellular drug level [89]. FOXM1 inhibitors, in combination with 5-FU, reduce colony formation, decrease cancer cell migration, and induce caspase-dependent apoptosis in colon and cholangiocarcinoma cancer cell lines [64,65].

b. Cytarabine is another pyrimidine analog used mainly in hematologic malignancies such as acute myeloid leukemia (AML). Cytarabine and anthracycline are the standard treatments for pediatric patients and medically fit adults with AML. Patients requiring more than one cycle of chemotherapy to achieve disease remission had worse survival outcomes [66]. Chemotherapy resistance correlated with higher nuclear FOXM1 expression in post-treatment bone marrow samples [66]. FOXM-1-overexpressed AML cells were resistant to standard chemotherapy in both in vitro and AML mouse models. Also, FOXM1 inhibition re-sensitizes resistant AML cells to cytarabine therapy. As a result, FOXM1 inhibitors can be studied concurrently or before standard AML chemotherapy to enhance treatment efficacy and restore drug sensitivity [66]

### 1.2. FOXM1 Inhibitors in Combination with Targeted Cancer Therapy

#### 1.2.1. Combination with Hormonal Therapies

a. Endocrine therapy in breast cancer: Estrogen or progesterone receptors are expressed in about 70% of breast cancers. Ligand-dependent pathway activation leads to the expression of multiple genes involved in cell proliferation and migration [90]. Aromatase inhibitors, estrogen receptor modulators, or antagonists showed survival benefits in early- and advanced-stage, hormone-positive breast cancers. However, on- and off-target acquired resistance have halted the durable therapeutic benefits. Intrinsic mutations in estrogen receptors contribute to the on-target resistance [90]. On the other hand, concurrent alterations in alternative oncogenic pathways, such as the PI3K-MTOR pathway, contribute to the off-target acquired resistance [90]. Another off-target resistance mechanism is FOXM1 overexpression. FOXM1 regulates the expression of the estrogen receptor α (ERα) in a positive feedback loop [91,92]. FOXM1 overexpression mediates endocrine resistance through the upregulation of the drug target and likely through the regulation of other cell cycle proteins, including cyclin D1 [54,92]. Moreover, FOXM1 promotes the expansion of resistant cancer stem cells and increases the expression of ABC transporters responsible for drug efflux [54]. In addition, the activated MAPK in tamoxifen-resistant breast cancer cells phosphorylates FOXM1 and induces its nuclear translocation and transcriptional activity [54]. Hence, FOXM1 inhibitors counteract the resistance encountered by hormonal therapies and prolong their therapeutic advantage [54].

b. Endocrine therapy in prostate cancer (PCa): Androgen (AR) signaling pathway drives proliferation and invasion in prostate cancer. Medical castration is the standard treatment for PCa. However, the chronic use of antiandrogens contributes to the development of castration-resistant PCa that is resistant to androgen therapy but still indirectly dependent on the AR signaling pathway [93]. The dependency on the androgen pathway occurs due to amplification of or mutations in the AR gene, which subsequently alters the drug target [93]. Also, FOXM1 is repeatedly overexpressed in resistant and metastatic PCa and plays a role in hormonal therapy resistance [67,68,94]. FOXM1 accelerates the development of PCa and induces tumor growth in mouse models [94]. FOXM1 regulates the transcription of androgen and androgen-responsive genes such as KLK2 and PSA [67,68]. Interestingly, FOXM1’s role in PSA expression dominates over AR in androgen refractory PCa [68]. Moreover, FOXM1 collaborates with AR to initiate and regulate DNA replication by controlling the expression of the CDC6 gene [67]. The combination therapy of antiandrogens and Siomycin A synergistically suppresses cell proliferation in androgen-sensitive and refractory prostate cancer cell lines [67,68].

#### 1.2.2. Combination with Reactive Oxygen Species (ROS) Inducers

ROS or free radicals are highly active molecules. They are produced in the mitochondria during energy (ATP) generation. Because cancer cells are metabolically active due to uncontrolled proliferation, they generate a higher level of ROS. Extremely elevated levels of ROS lead to the oxidation of important cell components like proteins, lipids, and DNA. This oxidative stress can drive cells to apoptosis. Therefore, cancer cells have an efficient system to maintain redox hemostasis. The efficient system of antioxidants consisting of glutathione, peroxiredoxin, thioredoxin, superoxide dismutase, and catalase induces redox status and protects cancer cells from oxidative stress. The increase in ROS production or inhibition in antioxidant enzymes could tip this balance [95]. ROS inducers are effective against tumors. However, they inflict dose-limiting side effects on normal tissue. Therefore, combination therapy with other drugs could synergistically induce anti-tumor activity with the use of lower drug doses. Interestingly, FOXM1 increases the expression of multiple antioxidant enzymes and effectively protects cells from oxidative stress related to ROS production [96]. Moreover, we recently demonstrated that FOXM1 can translocate into mitochondria, where it regulates oxidative phosphorylation [19]. Collectively, FOXM1 inhibitors and ROS inducers like PEITC or 2-methoxyestradiol exaggerate cellular oxidative stress and synergistically induce cellular death [69]. This combination therapy further shows efficacy in tumor xenografts [69].

#### 1.2.3. Combination with Poly (ADP-Ribose) Polymerase ½ (PARP ½) Inhibitors

PARP proteins play an essential role in base excision repair during single-strand DNA breaks (SSBs). Unfixed SSBs lead to more deleterious double-strand DNA breaks (DSBs), which are subsequently handled by homologous recombination (HR) [97]. HR is one of the two major DSB repair pathways. It is a complex process requiring a homologous template to repair DNA during the S and G2 phases. BRCA 1/BRCA2 proteins participate in HR repair. The inherited heterozygous deficiency of BRCA1 or BRCA2 increases the risk of breast, ovarian, and other cancers due to aberrant DNA repair. Cancer cells can survive a single alteration in BRCA1 or BRCA2 and subsequently adopt a backup DNA repair pathway. However, the addition of SSB repair inhibition drives cancer cells to death or so-called synthetic lethality [97]. Therefore, PARP inhibitors showed significant efficacy in HR-deficient tumors. However, to expand the therapeutic indications of PAPR inhibitors, different combinations were tested to induce synthetic lethality in HR-proficient tumors as well. The FOXM1 inhibitor, FDI-6, decreases the expression of PARP, BRCA1, and other DNA damage repair proteins and is thought to induce synthetic lethality in combination with other DNA repair protein inhibitors [55]. Indeed, FDI-6 synergized with Olaparib (a PARP inhibitor) and overcame Olaparib’s acquired resistance in pancreatic and triple-negative breast cancers [55,56]. The combination inhibited cell cycle progression and enhanced Olaparib’s DNA damage [55,56].

#### 1.2.4. Combination with Angiogenesis Inhibitors

Tumor angiogenesis is a vital process in tumor growth and proliferation. It is mediated mainly by hypoxia and the regulation of the transcription factor, Hypoxia-Inducible factor 1 (HIF-1) [98]. In a low-oxygen setting, HIF-1 activates the expression of the vascular endothelial growth factor (VEGF) and its receptors (VEGFRs). Angiogenesis inhibitors such as VEGFR2 monoclonal antibodies and tyrosine kinase inhibitors are currently in the clinic for the treatment of different solid tumors. These drugs are not without significant side effects, such as hypertension, thromboembolism, and hemorrhage. Moreover, tumor response is usually partial and short-lived. The mechanism of acquired resistance is largely unknown. However, the upregulation of HIF-1 upon VEGF inhibition could lead to VEGF-independent angiogenesis [98]. On the other hand, it is known that HIF-1 transcriptionally activates FOXM1 expression [99,100], and FOXM1 transcriptionally activates tumor angiogenesis through the upregulation of vascular endothelial growth factor (VEGF) and its receptor, VEGFR2 [101]. Therefore, FOXM1 can directly or indirectly be a culprit in VEGF inhibitor resistance. The concurrent inhibition of FOXM1 and VEGFR can provide greater inhibition of the angiogenesis pathway and decrease the dose of VEGF inhibitors. Indeed, the combination of a FOXM1 inhibitor with an anti-VEGFR2 monoclonal antibody improved tumor control compared to a single agent in hepatocellular carcinoma (HCC) tumor xenografts and provided a novel approach for the treatment of advanced HCC [70].

#### 1.2.5. Combination with a BCL2 Inhibitor (Venetoclax)

The mitochondrial BCL2 family proteins regulate the intrinsic apoptotic pathway. They are divided into four groups based on their structure and function. The anti-apoptotic proteins are BCL2, BCLxl, and MCL1. They bind directly to the pro-apoptotic proteins BAX and BAK. Proapoptotic signals induce BAX and BAK oligomerization and release cytochrome C from the mitochondria. BAX and BAK activation are controlled by pro-apoptotic activators such as BID, PUMA, and BIM. The fourth group of proteins, such as BAD and NOXA, cannot initiate apoptosis. However, they bind to anti-apoptotic proteins and suppress their function. BCL2 is overexpressed in different hematologic malignancies and is associated with chemotherapy resistance. Venetoclax is a highly selective BCL2 inhibitor, and it is a promising small molecule currently in the clinic for the treatment of CLL, AML, and other hematologic malignancies. One reported mechanism of venetoclax resistance is the upregulation of other antiapoptotic proteins like BCLxl and MCL1 [102]. The concurrent treatment of multiple antiapoptotic inhibitors may re-sensitize the cells to apoptosis. FOXM1 inhibition downregulates the expression of BCL2 and BCLxl and triggers caspase-mediated cell apoptosis [57,103]. Moreover, FOXM1 inhibition in AML cell lines upregulates multiple genes in the Homeobox A cluster (HOXA) family. HOXA overexpression positively correlates with venetoclax sensitivity in cancer cells [57,104]. Therefore, Chesnokov et al. proposed a novel combination therapy of RCM-1 and venetoclax to overcome venetoclax resistance and disrupt the FOXM1-AKT feedback loop in AML [57].

### 1.3. FOXM1 Inhibitors in Combination with Cell Cycle Inhibitors

FOXM1 expression varies during the cell cycle and is strictly controlled by different oncoproteins like cMYC [35]. Upon oncogenic stimulation, FOXM1 accumulates in the cytoplasm during the G1 to late S phases. It then undergoes phosphorylation and activation by other kinases like RAF/MEK/MAPK and cyclin D/CDK4/6 complex [34,35]. FOXM1 transcriptionally activates the expression of several cell cycle regulatory genes during the S phase. It induces the expression of Cdc25A phosphatase and diminishes the nuclear accumulation of CDK inhibitor proteins p21^Cip1^ (p21) and p27^Kip1^ [4,105]. During the G2/M transition, FOXM1 induces the transcription of Cdc25B phosphatase, Aurora B kinase, Survivin, and Polo-Like Kinase 1, the latter of which phosphorylates substrates essential for mitosis execution [5,106]. Additionally, FOXM1 increases cyclin B1 expression, which, in combination with CDK1, promotes cell entry into and out of the M phase. Late in the M phase, FOXM1 undergoes ubiquitin-mediated destruction and proteasomal degradation to halt cell cycle progression [34,35]. It is known that the loss of FOXM1 in cancer cells leads to mitotic spindle dysregulation and mitotic catastrophe [75,107,108]. The combination of FOXM1 inhibitors with other mitotic protein inhibitors is an attractive and promising therapeutic strategy.

#### 1.3.1. Combination with Aurora Kinase A (AURKA) Inhibitors

Yang et al. uncovered a positive feedback loop between FOXM1 and Aurora Kinase A (AURKA). FOXM1 recruits AURKA to transactivate FOXM1 target genes. On the other hand, AURKA binds to the FOXM1 promoter and induces its expression [71]. AURKAs are nuclear proteins and essential for proper cell division. They are located around the centrosome and microtubule, promoting bipolar microtubule spindle assembly and cell transition from G2 to M. Monotherapy with AURKA inhibitors such as Alisertib failed clinically to show significant anti-tumor activity in different tumors [109]. The mechanisms of resistance to AURKA inhibitors are largely unknown. However, Yang et al. proposed that the positive FOXM1-AURKA feedback loop could partially contribute to drug resistance. Accordingly, the authors discovered a synergistic activity of FOXM1 and AURKA inhibitors in vitro and in breast cancer mouse xenografts [71].

#### 1.3.2. Combination with Polo-like Kinase 1 (PLK1) Inhibitors

Similarly, Yu et al. revealed another feedback loop between FOXM1 and another mitotic protein, Polo-Like kinase 1 (PLK1) [72]. The expression of PLK1 is governed by FOXM1, and reciprocally, PLK1 also regulates the expression of FOXM1. PLK1 has an essential role in cell entry into mitosis through the phosphorylation and activation of cdc25C, which subsequently activates cyclin B. Also, PLK1, collectively with AURKA and Bora, degrades CDK1 inhibitors. Multiple PLK1 inhibitors have been discovered in the lab, and a few, such as volasertib, made it to clinical trials. Most PLK1 inhibitors lack specificity and have dose-limiting side effects in addition to the modest anti-tumor response seen in clinical trials [110,111]. To potentiate the anti-tumor effect and decrease toxicity, combination therapies are currently under investigation. Yu et al. found a modest response to PLK1 inhibitors in diffuse large B-cell lymphoma (DLBCL) cancer cells. Moreover, PLK1 inhibitors led to FOXM1 overexpression. Therefore, a linear inhibition of FOXM1 and PLK1 is a rational combination and has synergistically enhanced apoptosis in DLBCL cells. This combination can be potentially studied in other solid tumors where the FOXM1-PLK1 axis is known to be activated [72]. Consequently, there is a strong rationale for combining FOXM1 inhibitors with other mitotic inhibitors because of the crucial role of FOXM1 in regulating the G2/M cell transition and proper cell division.

#### 1.3.3. Combination with Cyclin-Dependent Kinases 4 and 6 (CDK4/6) Inhibitors

The CDK4/6 and cyclin D complex phosphorylates retinoblastoma (RB) protein and disassociates it from the transcription factor E2F. Subsequently, E2F increases the transcription of multiple target genes, including cyclin E genes, facilitating the G1/S phase transition. Also, CDK 4/6 phosphorylates and activates FOXM1 during the G1/S phase. This step is essential to execute FOXM1 nuclear translocation and transcriptional activity during the G2/M phase. Cell proliferation is strictly regulated by the CDK inhibitor proteins, the INK4 family, which bind to and inhibit CDK4/6 activity. Genetic alterations in G1/S cell cycle genes are frequent in cancer, for example, CDK4/6 and CCND1 amplifications, CDKN2A/2B loss, and hyperactivated mitogenic signaling pathways such as MAPK, β-catenin, and others [112]. Hence, targeting the cell cycle transition at G1 can be a successful strategy in cancer treatment. Selective CDK4/6 inhibitors are currently FDA-approved for hormone-positive and HER2-negative advanced and metastatic breast cancers. CDK4/6 inhibitors are also in use in multiple clinical trials for other cancers. CDK4/6 inhibitors induce cell senescence and growth arrest at G1. However, monotherapy with CDK4/6 inhibitors can face early and acquired resistance due to the switch of cell cycle dependency to other kinases, such as CDK2 [112]. Therefore, most of the available clinical trials are investigating the use of different synergistic combinations to mitigate the shift of cell survival dependency or to change cell fate from senescence to apoptosis in G1-arrested cells. The combination of FOXM1 and CDK4/6 inhibitors can further impair FOXM1’s role in DNA repair, inhibit cell cycle progression, and induce apoptosis [58,112]. Indeed, this combination showed a synergistic effect in hormone-positive breast cancer cell lines. It is an avenue to expand the use of CDK4/6 inhibitors in the treatment of breast cancer [58].

### 1.4. FOXM1 Inhibitors Decrease Immune Evasion and Synergize with Immunotherapy

Harnessing the immune system to attack tumor cells was a therapeutic goal for many years until the breakthrough of CTLA4 inhibitors and subsequently PDL1 inhibitors. Immunotherapy is currently the first line of treatment for many metastatic, locally advanced, and hypermutated tumors like melanoma, non-small-cell lung cancer, and others. One of the challenges of expanding the label to other solid tumors, especially sarcomas, where immunotherapy repeatedly failed to show an objective response in most subtypes, is the immune-suppressive tumor microenvironment in relatively “cold” tumors [113]. Moreover, immune tolerance has been reported in “hot” tumors after monotherapy with immune checkpoint inhibitors (ICIs) [114]. Combination therapies have been evaluated to enhance the immune response and maintain durable disease control. PD-1 is a T-cell surface marker that binds to its ligand (PDL1) on tumor cells and exhibits an inhibitory effect on T cells. This interaction leads to tumor immune evasion. It has been shown that FOXM1 binds to the promoter of the PDL1 gene and induces its expression [59]. It has also been demonstrated that in addition to high levels of FOXM1 in tumor cells, FOXM1 is also highly expressed in immunosuppressive tumor-associated macrophages (TAMs) [24]. Targeting FOXM1 by TST or small molecules such as RCM-1 downregulates PDL1 levels and subsequently enhances CD3+ T-cell immune filtration [59,60]. Also, the conditional knockdown of FOXM1 in TAMs inhibits tumorigenesis and induces pro-inflammatory cytokines [24]. FOXM1 inhibitors combined with immunotherapy like PD-1 inhibitors synergistically increase tumor apoptosis with tolerated immune-related side effects in syngeneic mouse models [59,60]. This combination therapy is a promising strategy to enhance the anti-tumor immunity in cold tumors and a potential path to expand the use of immunotherapy.

## 2. Conclusions and Future Directions

**Figure 2:**
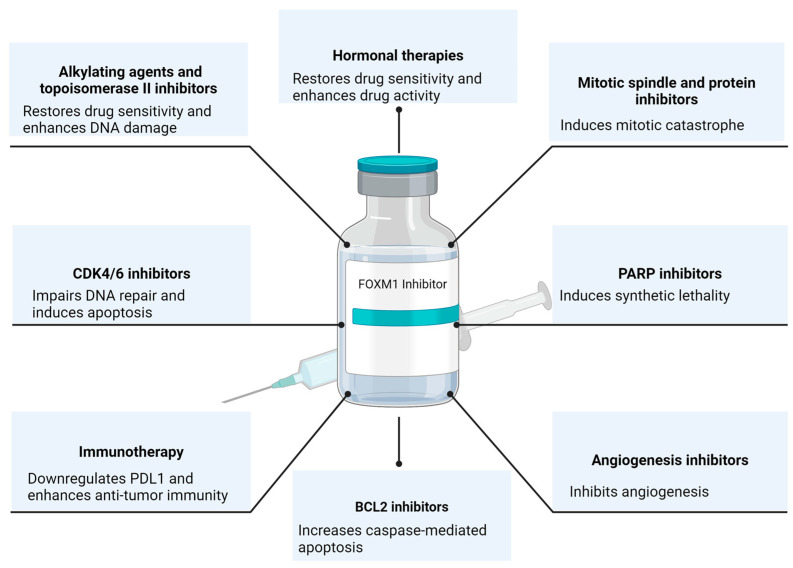
The Rational of FOXM1 Combination Therapies. Created with BioRender.com (accessed on 11 January 2024).

The essential role of FOXM1 in tumorigenesis has been established and verified in many different cancers. FOXM1 inhibition has been proposed as an attractive therapeutic target, and an extensive body of literature supports the therapeutic benefits of inhibiting the transcriptional activity of FOXM1. Hence, transitioning FOXM1 inhibitors to clinical trials is a long-awaited step. However, the challenges of suppressing a transcription factor with the risks of untoward off-target side effects have halted its progress to clinical studies. While the newer FOXM1-targeted inhibitors were found to be specific in the lab, more extensive pre-clinical work is still needed to confirm their specificity and safety in animal models. Also, more studies are required to optimize drug delivery to improve bioavailability and decrease side effects. Nevertheless, we reviewed here the previously studied combination therapies with FOXM1 inhibitors (Figure 2). In relapse regimens, there is a strong rationale for combining FOXM1 inhibitors with cytotoxic chemotherapy or hormonal therapy to re-sensitize tumors and maintain a durable response. FOXM1 inhibitors can also be utilized as a maintenance monotherapy after chemotherapy to prevent relapse through their functions in suppressing stemness in cancer cells. In the upfront regimens, FOXM1 inhibitors can induce synthetic lethality with PARP inhibitors or cause mitotic catastrophe with mitotic inhibitors. Extracting from the clinical experience of other small-molecule inhibitors, the incomplete suppression of the oncogenic pathway or the shift of cell survival dependency onto another pathway suggests the need for dual-targeted therapy to optimize the response, overcome acquired resistance, and decrease drug dosing. Furthermore, FOXM1 inhibitors, through their role in PDL1 downregulation, can sensitize tumors to immunotherapy and cellular therapy. In conclusion, FOXM1 inhibitors synergize with different traditional and targeted anti-cancer therapies. The avenue where FOXM1 inhibitors can be utilized is only expanding with additional research emerging and highlighting the unique role of FOXM1 in different aspects of tumor development and progression.

## Figures and Tables

**Table 1:** Summary of drug combinations with FOXM1 inhibitors.

FOXM1 Inhibitor	Drug Combination	Cancer Type	Treatment-Naïve vs. -Resistant	Rationale	References
Specific FOXM1 Inhibitors					
RCM-1	Vincristine	Rhabdomyosarcoma, breast cancer, melanoma, lung, pancreatic and prostate adenocarcinoma	Naïve	Delay in mitosis, decrease in β-catenin protein	[47,50]
Liposomal FOXM1 aptamer	Doxorubicin	Breast cancer	Naïve and resistant	Apoptosis induction	[53]
p19^ARF^ 26-44 peptide	4-OH-TAM (SERM)	Hormone-positive breast cancer	Resistant	Downregulation of FOXM1 target genes, downregulation of ABCG2, CDC42, and RhoB	[54]
FDI-6	Olaparib (PARP inhibitor)	Pancreatic and triple-negative breast cancer	Naïve	Decrease in PARP, BRCA1, and other DDR proteins	[55,56]
RCM-1	Venetoclax (BCL2 inhibitor)	AML	Naïve	Downregulation of BCLxl and suppression of FOXM-1-AKT feedback loop	[57]
NB73 or NB115	Palbociclib, Ribociclib or Abemaciclib (CDK4/6 inhibitor)	Hormone-positive breast cancer	Naïve	Suppression in cell cycle progression and apoptosis induction	[58]
RCM-1	4-1BB and an anti-PD-1 inhibitor (immunotherapy)	Non-small-cell lung cancer (NSCLC), colon cancer	Naïve	Downregulation in PDL1 expression	[59,60]
Other FOXM1 Inhibiting Drugs					
Bortezomib	Temozolomide	High-grade glioma	Resistant	Suppression of FOXM1–Survivin axis	[61]
Thiostrepton	Paclitaxel	Pancreatic cancer, ovarian cancer	Naïve and resistant	Downregulation of Prohibin1, CCNB1, and CDC25B, decrease in ABCA2 expression	[62,63]
Thiostrepton	Fluorouracil	Colon cancer	Resistant	Decrease in TYMS expression, decrease in ABCC10 expression.	[64]
Siomycin A	Fluorouracil	Cholangiocarcinoma	Resistant	Decrease in TYMS expression	[65]
Ixazomib	Cytarabine	AML	Resistant	Apoptosis induction	[66]
Siomycin A	Bicalutamide (Antiandrogen)	Androgen-sensitive and refractory prostate cancer	Naïve and resistant	Downregulation in CDC6 gene and androgen-responsive genes; PSA and KLK2	[67,68]
Bortezomib or thiostrepton	PEITC or 2-methoxyestradiol (ROS inducers)	Pancreatic, liver, and breast cancers	Naïve	Downregulation of antioxidant enzymes and induction of oxidative stress	[69]
Carfilzomib	DC101 (VEGFR2 monoclonal antibody)	Hepatocellular carcinoma	Naïve	G2/M cell cycle arrest and suppression of proliferation	[70]
Thiostrepton	AKI603 (AURKA inhibitor)	Triple-negative breast cancer	Naïve	Suppression of cancer stem cells and FOXM1 target proteins	[71]
Thiostrepton	Volasertib (PLK1 inhibitor)	DLBCL	Naïve	Suppression of FOXM1 target proteins such as cyclin B1 and CHK1	[72]
